# Functional Alteration of Cerebello–Cerebral Coupling in an Experimental Mouse Model of Parkinson’s Disease

**DOI:** 10.1093/cercor/bhy346

**Published:** 2019-01-31

**Authors:** Fabien Menardy, Andrés Pablo Varani, Adèle Combes, Clément Léna, Daniela Popa

**Affiliations:** Neurophysiology of Brain Circuits Team, Institut de Biologie de l’Ecole Normale Supérieure (IBENS), Ecole Normale Supérieure, CNRS, INSERM, PSL Research University, Paris, France

**Keywords:** 6-OHDA, cerebellum, electrophysiology, motor cortex, optogenetics, Parkinson’s disease

## Abstract

In Parkinson’s disease, the degeneration of the midbrain dopaminergic neurons is consistently associated with modified metabolic activity in the cerebellum. Here we examined the functional reorganization taking place in the cerebello–cerebral circuit in a murine model of Parkinson’s disease with 6-OHDA lesion of midbrain dopaminergic neurons. Cerebellar optogenetic stimulations evoked similar movements in control and lesioned mice, suggesting a normal coupling of cerebellum to the motor effectors after the lesion. In freely moving animals, the firing rate in the primary motor cortex was decreased after the lesion, while cerebellar nuclei neurons showed an increased firing rate. This increase may result from reduced inhibitory Purkinje cells inputs, since a population of slow and irregular Purkinje cells was observed in the cerebellar hemispheres of lesioned animals. Moreover, cerebellar stimulations generated smaller electrocortical responses in the motor cortex of lesioned animals suggesting a weaker cerebello–cerebral coupling. Overall these results indicate the presence of functional changes in the cerebello–cerebral circuit, but their ability to correct cortical dysfunction may be limited due to functional uncoupling between the cerebellum and cerebral cortex.

## Introduction

The cerebellum and the basal ganglia are 2 brain structures that provide the bulk of subcortical projections—via the thalamus—to the motor and premotor cortices and that form closed loops with these cortical areas (Alexander et al. 1986; Hoover and Strick 1999). Neurodegenerative disorders may affect more specifically one of the structures and less the other. A striking example is Parkinson’s disease (PD), one of the main neurodegenerative disorders initiated by the loss of dopaminergic neurons in the basal ganglia, leading to the cardinal motor symptoms of bradykinesia and rigidity accompanied often by resting tremor (Albin et al. 1989; Lees et al. 2009). As detailed below, the loss of these neurons has extensive functional consequences in the basal ganglia but also beyond, notably in the motor cortex and, as acknowledged more recently, in the cerebellum. It is currently unclear how these changes relate to another, in particular within the cerebellum and between the cerebellum and other areas.

The loss of striatal dopamine, a major modulator of striatal function (Surmeier et al. 2007), is first accompanied by functional changes within basal ganglia circuits and in the cortico–striato–thalamo–cortical loop (Blandini et al. 2000; Mallet et al. 2006; Gale et al. 2008; Lees et al. 2009). Resting-state neuroimaging studies have shown that dopaminergic depletion induces an increase in glucose metabolism in the globus pallidus, one of the inhibitory basal ganglia outputs, and in the thalamus, but a reduction in the premotor cortex and posterior parietal areas (Eidelberg et al. 1994; Ma et al. 2007; Peng et al. 2014). These changes are accompanied at rest by a decreased functional connectivity between the striatum, the thalamus and the sensorimotor cortex (Wu et al. 2009; Hacker et al. 2012; Helmich et al. 2012). Interestingly, increased functional connectivity between the anterior striatum and cortical areas, together with changes of metabolic activations in PD patients at rest or during motor tasks, argue for a reorganization of the circuits and could reflect compensatory mechanisms within and outside basal ganglia (Wu and Hallett 2005; Palmer et al. 2010; Wu et al. 2011; Wu and Hallett 2013). The changes in neuronal activity and firing patterns underlying the metabolic changes are less extensively established but indicate an increased firing rate and synchronization within subthalamic nucleus and globus pallidus pars interna (Bergman et al. 1994; Vila et al. 2000; Lin et al. 2008). The changes of firing within basal ganglia lead to an aberrant over-inhibition of the basal ganglia thalamic recipient nuclei and thus a decreased motor cortex activity (Valls-Sole and Valldeoriola 2002; DeLong and Wichmann 2007).

Noninvasive functional imaging studies provide strong evidence of a functional reorganization beyond the cortico–striato–thalamo–cortical pathway in the brain of PD patients described above. At rest, imaging of the brain activity has revealed a “default mode” network, a set of brain areas (including the cerebellum) that show increased coactivation in the absence of any focused cognitive activity (Raichle 2015). In healthy subjects and patients in the early stages of PD, the activity of the default-mode network is reduced during motor task execution while, in advanced PD, this task-related deactivation of the default-mode network is reduced (Spetsieris et al. 2015), and replaced by a specific pathological PD-related pattern, characterized by increased pallido–thalamic and pontine and cerebellar metabolic activities, and moderate reductions of metabolic activity in the premotor cortex, supplementary motor area, and parietal association regions (Huang et al. 2007; Ma et al. 2007; Eidelberg 2009). Changes in the cerebello–thalamo–cortical pathway in PD have therefore received an increased interest in the recent years (reviewed in Martinu and Monchi 2013; Wu and Hallett 2013). The cerebellum exhibits a marked hypermetabolism in PD patients (Ma et al. 2007; Eidelberg 2009; Helmich et al. 2012) accompanied with an increased connectivity between motor, parietal cortical areas and the cerebellum (Palmer et al. 2010; Wu et al. 2009; 2011). Movements in PD patients are accompanied with an increased metabolic activity in the cerebellum (Wu and Hallett 2005; Yu et al. 2007; Mure et al. 2011), and in early PD, recruitment of the cerebello–thalamo–cortical circuit positively correlates with the progression of motor features (Sen et al. 2010; Wu et al. 2011). Some of these changes could support compensatory mechanisms to the basal ganglia dysfunction (Bezard et al. 2003; Wu and Hallett 2013). However, the cerebello–thalamo–cortical pathway seems also involved in the genesis of resting tremors, and stimulation of this pathway is indeed a target for corrective treatment of PD tremor (Benabid et al. 1991; Timmermann et al. 2003; Helmich et al. 2011, 2012; Dirkx et al. 2017).

Most studies cited above report metabolic consumption rather than neuronal activity to infer cerebellar involvement in PD. The Purkinje neurons, which form its output station of the cerebellar cortex only use a small fraction of the neuronal signaling metabolism (Howarth et al. 2010) and are therefore not likely to contribute massively to the signal in imaging studies. Therefore, the changes in cerebellar output in PD remain largely unknown. Interestingly, changes in the coupling between the cerebellar and cerebral cortices could also take place in PD, as evidenced in patients by the weakening of the “cerebellar brain inhibition” (i.e., the suppression of M1-evoked electromyographic responses following a contralateral transcranial magnetic stimulation over the cerebellum) (Ni et al. 2010; Carrillo et al. 2013). This somewhat indirect evidence suggests a decreased excitability in the cerebello–thalamo–cortical pathway in PD disease. It is also currently unclear whether such decrease in cerebello–cortical ascending coupling is accompanied with changes in the descending cerebello–motor effectors coupling, as it has been reported for the motor cortex in PD patients and animal models (Ellaway et al. 1995; Viaro et al. 2011).

The goal of our study is to investigate the neurophysiological changes taking place in the cerebello–cortical circuit in PD, using a rodent model of the disease using the 6-OHDA (6-hydroxydopamine) toxin to lesion mesostriatal dopaminergic neurons. In this model, abnormal neuronal activities develop in the basal ganglia and in the motor cortex in the weeks following the lesion of dopaminergic systems (Bergman et al. 1994; Mallet et al. 2008; Degos et al. 2009; Parker et al. 2016). In order to explore the cerebellar functional connectivity, we performed electrophysiological recordings, and performed cerebellar cortex stimulations in mice specifically expressing channelrhodopsin 2 in Purkinje cells (Chaumont et al. 2013; Proville et al. 2014). This allowed us to probe cerebellum-evoked movements, examine the electrophysiological state of the cerebellum, and the functional coupling between the cerebellum and cerebral cortex.

## Material and Methods

### Animals

All animal procedures were performed in accordance with the recommendations contained in the European Community Council Directives. The animals were housed under standard conditions, on a 12 h light/dark cycle with ad libitum access to food and water. All experiments were performed in awake freely moving mice except the juxtacellular recordings obtained in anaesthetized L7-ChR2 adult (8 weeks and older) mice (25–35 g) (Chaumont et al. 2013).

### Dopaminergic Lesions

Surgical procedures were performed as described in (Francardo et al. 2011). Briefly, the animals were anesthetized (isoflurane 1.2% or ketamine 50 mg/kg and xylazine 10 mg/kg, i.p.) and pretreated with desipramine (25 mg/kg i.p.). Then, the animals were mounted in a stereotaxic frame (David Kopf Instruments, USA) and they received a 10-min unilateral injection of 1 μL of 6-OHDA (3.2 μg/μL free-base in a 0.02% ascorbic acid solution) or vehicle (SHAM condition: 1 μL ascorbic acid solution) in the medial forebrain bundle of the right or left hemisphere (from Bregma: −1.2 AP, 1.3 ML, −4.75 depth from dura). To prevent dehydration, the mice received sterile glucose–saline solution (50 mg/mL, 0.01 mL/g body weight, s.c. (Francardo et al. 2011)) immediately after surgery and twice a day during the first 2–3 weeks postsurgery.

### Histology

After recordings, the animals were given an overdose of pentobarbital (100 mg/kg, i.p.) and brains were removed from the skull after an intracardiac perfusion with paraformaldehyde (4%), stored in paraformaldehyde (4%) and then sectioned in a microtome at a thickness of 40 μm. Immunocytochemical labeling of TH (rabbit anti-TH antibody (Chemicon, Temecula, CA, USA) 1:1000) was performed in the striatal slices of sham-operated or 6-OHDA-lesioned mice to verify the extent of the lesion (Supplementary Fig. S1), using the protocols as in Francardo et al. (2011).

### Behavioral Test and Analysis

**Figure 1.**
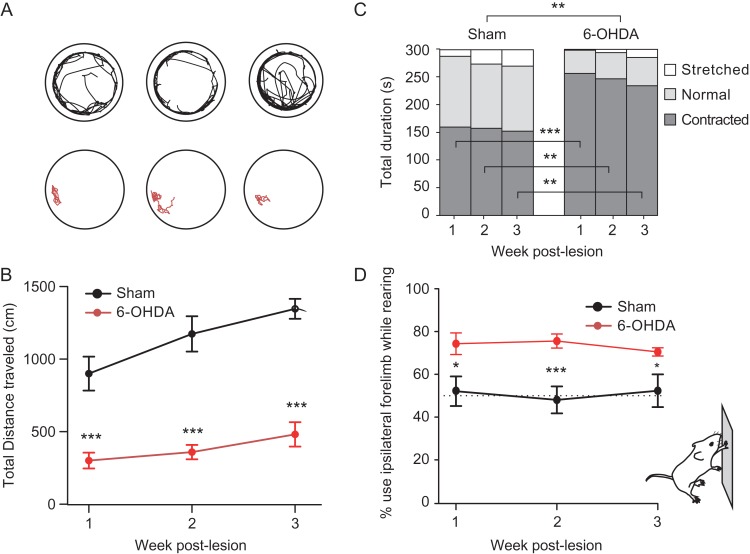
Motor impact of dopaminergic lesion. (*A*) Example openfield traces (5 min) from sham-operated (upper line, black), and 6-OHDA-lesioned (lower line, red) mice. (*B*) evolution of total distance in 5 min sessions over the 3 weeks postoperation. (*C*) Distribution of postural states in sham-operated and 6-OHDA-lesioned animals. (*D*) Percentage wall contacts while standing where the forelimb ipsilateral to the lesion was used for body support over the 3 weeks postlesion. Statistics correspond to one way ANOVA (panels *B*, *D* and between bars in panel *C*) and repeated-measure ANOVA (between Sham and 6-OHDA panel *C*); see text for panels *B*,* D*); *P < 0.05, **P < 0.01, ***P < 0.001.

To evaluate the motor deficits following dopaminergic depletion, we recorded the locomotor activity in the open field test, and the asymmetry in the use of limbs to stand against the walls in a cylinder during 3 weeks in sham-operated and lesioned mice (Fig. 1). These tests have been respectively associated with reduced locomotion and locomotion speed and reduced limb contralateral to the lesion in models of PD (Iancu et al. 2005; Glajch et al. 2012; Heuer et al. 2012; Carvalho et al. 2013). We also recorded the head movement with accelerometers, in order to obtain high-resolution measures of the head motor activity.

#### Open-Field Test

To evaluate locomotor activity in 6-OHDA-lesioned and sham-operated mice, mice were placed in a 38 cm diameter circular arena (Noldus, Netherlands) and recorded by video from above. The position of the center of gravity of the mice was tracked with Ethovision 11 (Noldus, Netherlands) and the distance traveled, velocity, periods of inactivity and body elongation were assessed for a 5 min period. Inactive periods were defined as periods of time during which the average pixel change of the entire video image was less than 0.8% (from one video frame to another one). To evaluate the posture of the animals, we used Ethovision body elongation measures (Ethovision, Nodlus, Netherland). Briefly, the shape of body is measured using the coordinates of the subject’s contour (x and y) and subjects’ center ($x_{c}$ and $y_{c}$):

$$Elongation=\frac{\sqrt{{\left(\sum_{x}{\left(x-x_{c}\right)}^{2}-\sum_{y}{\left(y-y_{c}\right)}^{2}\right)}^{2}-4{\left(\sum_{x,y}(x-x_{c})(y-y_{c})\right)}^{2}}}{\sum_{x}{\left(x-x_{c}\right)}^{2}-\sum_{y}{\left(y-y_{c}\right)}^{2}\;}$$

Elongation is expressed as a percentage ranging from 0 (when the subject’s shape is perfectly circular) to 100% (when the subject’s shape is a line). According to the body elongation percentage we defined 3 possible states as the following: stretched, if the body elongation percentage is greater than 70%, contracted for a percentage is lower than 50%, and Normal for a value between contracted and stretched threshold.

#### Cylinder Test

To assess lateralized motor deficits in mice with unilateral 6-OHDA-lesions compared to sham-operated mice, we placed them during 3 min in a 10 cm diameter vertical glass cylinder while monitoring the rearing events using video monitoring. No habituation was allowed before video recording. Weight-bearing wall contacts made by each forelimb on the cylinder wall were scored. Limb use was expressed in terms of the percentage of the number of wall contacts with impaired forelimb (i.e., contralateral to the lesion) relative to the total number of times the mouse touched the wall with one or both of the forelimbs; changes in limb use during a standing episode were counted as separate touch events.

#### Accelerometer Recordings

Head movements were recorded using a digital microelectromechanical sensor combining a linear 3D accelerometer and a 3D gyroscope (Ivensense, Mpu-9150). We used a custom printed circuit carrying the surface components (sensor, resistors and capacitors) and the power and data exchange wires (Dugue et al. 2017). To communicate with the sensor, we use an I2C interface controlled via the USB port by a program in the LabVIEW software to synchronize the signal from the accelerometer with the electrophysiological system and optogenetic stimulations. Stimulation-triggered averages were computed in R. Periods of head immobility were defined as periods of at least of 1 s duration with head rotation speed always below 20 deg/s duration.

**Figure 2.**
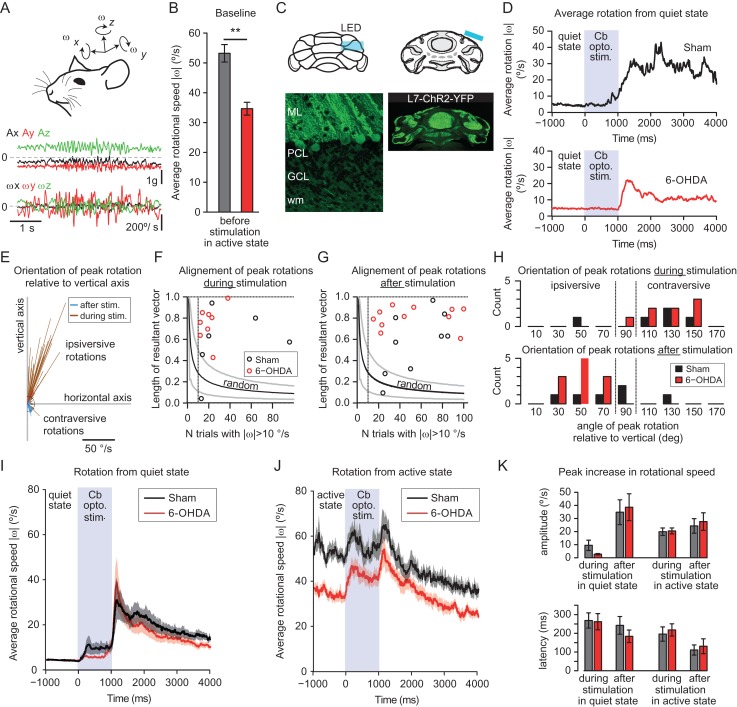
Head movement evoked by hemisphere cerebellar stimulation in mice expressing ChR2 in Purkinje cells 3 weeks after a 6-OHDA lesion or a sham operation. (*A*) Example trace of instantaneous 3D acceleration (Ax, Ay, Az) and 3D rotation (ωx, ωy, ωz) recorded from the head-bound inertial sensor. (*B*) Average rotational speed in sham-operated and 6-OHDA-lesioned animals in the active state (|ω|>20 deg/s); note the bradykinesia on head movements. (*C*) Scheme of the optogenetic stimulation performed with an implanted micro-LED above Crus I hemisphere. (*D*) Example of average trace for single mice where the stimulation started while the animal was immobile in the second that preceded the stimulation onset. Grayed area indicate periods of time with a cerebellar optogenetic stimulation. (*E*) Orientation of the rotation axis relative to vertical within one recording session. Each line represents a single trial, the length of the segment represents to the peak rotational speed and its direction indicates the orientation relative to the vertical (direction of the gravity). Blue lines (mostly in the lower right quadrant) represent the peak rotations during the optogenetic stimulation, while the brown lines represent the peak rotations after the end of the optogenetic stimulation. The dotted circle corresponds to 10 deg/s (which is typical of resting state). Note that most rotations after the end of stimulations are oriented in the top-right quadrant corresponding to ipsiversive rotations. (*F*, *G*) Alignment of peak rotation axes during (*F*) and after (*G*) the stimulation. The alignment is quantified using the length of the resultant vector (see Material and Methods), obtained by summing unity vectors in the direction of the axis of peak rotation for all the trials with a peak rotational speed above 10 deg/s and dividing the result by the number of vectors summed. The plot indicates the resultant length as a function of the number of vectors summed. Each point corresponds to one animal (black: sham-operated mice, red: 6-OHDA lesioned mice). The black and gray lines correspond respectively to the average and to the 5–95% confidence interval for the resultant length obtained from random distribution of direction. Note that all 6-OHDA-lesioned animals and most sham-operated (but 3) show a strong alignment of the axis of the evoked rotation. (*H*) Distribution of the orientation of the resulting vector relative to the vertical axis (direction of gravity); only mice with good alignment of their rotation axis (resulting length>0.4) are plotted. (*I*) Average rotational speed while stimulating the cerebellar cortex after a 1 s period of head immobility. Note the large movements evoked at the end of the stimulation compared to the movements observed during the stimulation. Data from 10 sham-operated and 12 6-OHDA-lesioned mice. Traces are mean ± SD. The grayed area represents the period of time with cerebellar optogenetic stimulation. (*J*) Average rotational speed while stimulating the cerebellar cortex with movements occurring in the second that preceded the stimulation. Note the rotational speed increases during the stimulation are larger than observed for stimulation in quiet states (panel *I*). Traces are mean ± SD. (*K*) quantification of the movements (peak rotational speed and latency to peak) observed during and after the stimulation.

The head rotations were examined in stimulation-triggered average of the rotational speed (Fig. 2), and the rotations were analyzed at the time of peak rotational speed. Rotational speed refers to the Euclidian norm of the angular velocity vector. To examine the consistency of the stimulation-induced rotations, we used the resultant vector ***v*** obtained by summing unity vectors in the direction of the single trials’ rotation velocity vectors at the time of the peak rotational speed, and by dividing the sum by the number of unity vectors summed:

$$v=\frac{1}{N}\overset{N}{\underset{i,\;|\omega_{i}^{peak}|\;>\;10deg/s\;}{\sum }}\frac{\omega_{i}^{peak}}{\left|\omega_{i}^{peak}\;\right|}$$

If individual unity vectors are aligned, the length of the resultant vector is 1; if unity vectors are randomly oriented, it tends progressively toward 0 when the number of unity vectors summed increases (see lines in Fig. 2*F*,*G*). To identify animals with consistent stimulation-induced rotations, we computed the distribution of length of resultant vectors obtained from 10 000 samples of *N* random directions unity vectors, *N* ranging from 1 to 100; this allowed us to compute the expected average length and 2.5–97.5% confidence interval for random distribution; values of resultant length falling outside of these interval are considered to indicate a bias in the distribution of directions (Fig. 2*F*,*G*).

### Optogenetic Stimulations in Awake L7-ChR2 Mice

To stimulate Purkinje cells, 3 micro LEDs (1.6×0.6 mm^2^ right angle SMD chip LED lamp, Kingbrigth, USA) emitting blue light with a dominant Wavelength 470 nm were placed above Crus I of the cerebellum in order to cover most of its extent. A small craniotomy window (approximately 1.6 × 1.8 mm^2^) centered over Crus I (from Bregma: −6.5 AP, 3.5 ML) was performed and filled with agarose 2% above the dura to prevent damage of the cerebellar cortex. Then a small piece of coverslip glass was placed above the agarose and the LEDs were connected to insulated power wires, were glued to the glass and embedded in dental cement. During experiments, 1 s pulses of 8 mW/mm^2^ irradiance were delivered at 0.25 Hz.

### Recordings

Recordings in awake freely moving mice were performed in the 3 weeks after the sham operation or the 6-OHDA lesion of the nigrostriatal pathway. Recordings were performed using an acquisition system with 32 channels (sampling rate 25 kHz; Tucker Davis Technology System 3, Tucker-Davis Technologies, Alachua, FL, USA) as described in (Popa et al. 2013). The cerebellar nuclei are preferentially sending projections to the contralateral meso-diencephalic regions (Teune et al. 2000), so recordings were performed in motor cortex M1 contralateral to the cerebellum and ipsilateral to the lesion.

#### Chronic Recordings

To record cells activity in the primary motor Cortex M1 and in the cerebellar nuclei we used bundle of electrodes consisting of nichrome wire (0.005 inches diameter, Kanthal RO-800) folded and twisted into bundles of 6–8 electrodes. To protect these bundles and ensure a good electrode placement in the brain, they were then inserted in metal cannulas (8–10 mm length, 0.16–0.18 mm inner diameter, Coopers Needle Works Limited, UK) attached to an electrode interface board (EIB-16 or EIB-32; Neuralynx, Bozeman, MT, USA) by Loctite universal glue in a configuration allowing us to record motor cortex M1 (AP: 2,2; ML: 2,2; DV: 200–1000 μm) or the cerebellar nuclei (from Bregma: Fastigial: −6.0 AP, ±0.75 ML, −2.1 depth from dura; Interposed: −6.0 AP, ±1.5 ML, −2.1 depth from dura; Dentate: −6.2 AP, ±2.3 ML, −2.4 depth from dura).

**Figure 3.**
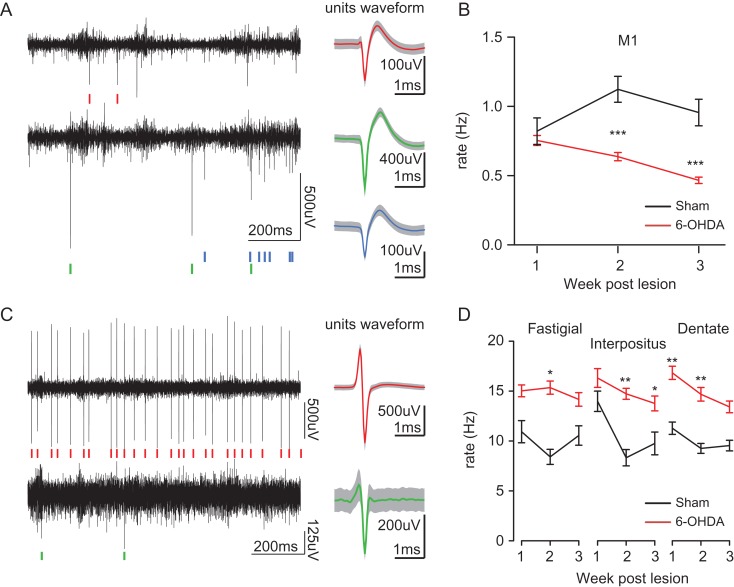
Neuronal activity in primary motor cortex (M1) and cerebellar nuclei. (*A*) Left: excerpt of traces and spikes (color bars) from the motor cortex; right: average waveform for the neurons sorted on the channels; gray lines indicate the standard deviation of the traces. (*B*) Evolution of motor cortex M1 firing in openfield across the 3 weeks postoperation. (*C*) Excerpts of traces and spikes from the crebellar nuclei (same organization as in panel *A*). (*D*) Average firing rate in the cerebellar nuclei. Repeated measure ANOVA (mouse as random factor) *P < 0.05, **P < 0.01, ***P < 0.001.

The microwires of each bundle were connected to the EIB with gold pins (Neuralynx, Bozeman, MT, USA). The entire EIB and its connections were secured in place by dental cement for protection. The electrodes were then cut to the desired length (extending 0.5 mm below tube tip). The impedance of every electrode was measured and the 1 kHz impedance was set to 200–500 kΩ using gold-plating (cyanure-free gold solution, Sifco, France). During the surgery, sham operation or 6-OHDA lesions were performed in mice under anesthesia, and then the skull and the dura were removed above motor cortex M1 cortices or cerebellar nuclei recording site. The electrode bundles were lowered into the brain and the ground was placed over the cerebellum. One week after the surgery, we started to record cellular activity in motor cortex M1 (layers 2–5) or cerebellar nuclei in freely moving mice placed in an openfield (Fig. 3). The signal was acquired by a headstage and amplifier from TDT (RZ2, RV2, Tucker-Davis Technologies, Alachua, FL, USA). The spike sorting was performed with Matlab (Mathworks, Natick, MA, USA) scripts based on *k*-means clustering on PCA of the spike waveforms (Paz et al. 2006). At the end of experiments, the electrodes’ positioning in the motor cortex M1 or cerebellar nuclei was verified by electrolytic lesions and compared to a brain atlas after brain slicing (Supplementary Fig. S1). The yield of recordings was rather stable over the recording period, but no statement is made whether the same cells were recorded across sessions.

#### Acute Recordings

**Figure 4.**
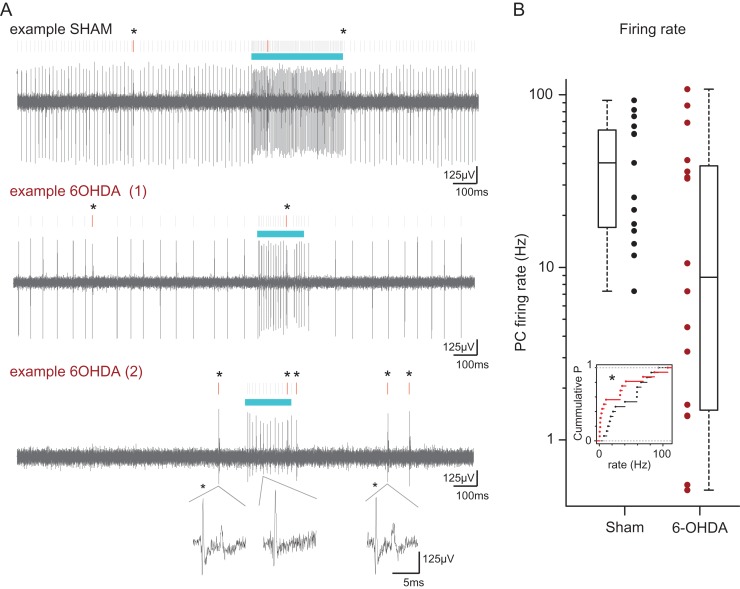
A pool of slow Purkinje cells in 6-OHDA-lesioned animals expressing the ChR2 in Purkinje cells. (*A*) Excerpts of traces of extracellular recording with a glass optrode of Purkinje cells at rest and during optogenetic stimulations; top: cell from a sham-operated animal; middle and bottom: cells from 6-OHDA-lesioned animals. In the bottom panel, the cell emits only complex spikes spontaneously. The blue bars signal the optogenetic stimulations; vertical bars signal spikes, red bars and stars signal complex spikes. Example of complex spikes and simple spikes traces are provided at the bottom. (*B*) Comparison of the spontaneous firing rate of cells recorded in sham-operated and 6-OHDA-lesioned animals; each point corresponds to a single cell. Inset: distribution of spontaneous firing rates; note the leftward shift of firing rates toward low values in 6-OHDA-lesioned animals. Kolmogorov Smirnov test: *P < 0.05.

In another series of experiments, we performed in vivo juxtacellular recordings of Purkinje cells in 6-OHDA-lesioned and sham-operated L7-ChR2 mice. Recordings were performed with a borosilicate glass pipette (2.5 μm tip diameter; 1.5 mm O.D. × 0.86 mm I.D.) containing an optical fiber connected to a laser in order to illuminate the cell at the tip of the pipette, in the weeks after the lesion of the nigrostriatal pathway under anesthesia (urethane: 1.6 g/kg i.p.); one mouse was recorded 13 days after the lesion (and provided only one Purkinje cell with a firing rate of 33 Hz), and all the others were between 19 and 22 days postlesion. To prevent illumination along the entire pipette, the major part of the glass pipette was blackened by immersion in a permanent black ink while a constant air pressure was applied to the pipette by an air pump to prevent it from becoming clogged. Then, the pipette was filled with physiological extracellular medium (in mM: NaCl 140, KCl 2,5, CaCl_2_ 1,6, MgCl_2_ 1,5 HEPES NaH2PO4 1,25 1.6 mMCa, 1.5 mM Mg, pH = 7.4) and placed in an electrode holder (QSW-B15P 64–0827, Warner Instrument, USA). The holder was connected to a custom amplifier in current clamp mode and an Axon Instruments Digidata 1200 Interface (Molecular Devices, USA). Optogenetic activation (varying in intensity and duration, from 1.16 to 5.63 mW/mm^2^ from pipette tip and 100–400 ms) were delivered by blue-light illumination (473 nm) through a thin optical fiber (105 μm Core, AFS105/125Y—Custom, Thorlabs, USA)—placed via a path in the electrode holder within the glass pipette next to the tip—and coupled to a Laser (CL-473-075 Laser and CL-2005 Laser Power Supply, CrystaLaser, USA). Purkinje cells were recorded from different sites in the cerebellar cortex and were identified by increased firing rate response to optogenetic stimulations (Fig. 4) and/or presence of complex spikes, identified help to the automatic template detection of waveforms (Spike 2, CED, Cambridge, UK); the complex spikes were characterized by reproducible, increased postspike deflection and waves in the field potential (spikelets) after the main spike (see examples in Fig. 4). No attempt was made to label the recorded cells by juxtacellular electroporation.

### Statistics

Values in the text and figures are mean ± standard error on the mean unless specified otherwise. Statistical significance was assessed by ANOVAs (or repeated-measure ANOVA when appropriate) followed by post hoc Student’s *t*-test. ANOVAs were performed on log-transformed values of the firing rate and of the median of the interspike intervals to normalize the shape of the distributions. Two statistics were used to study the structure of the cells’ discharge: the coefficient of variation of the interspike intervals, which broadly indicates discharge irregularity, and the presence of bursts (defined as a sequence of short interspike intervals with very rare expected occurrence if these intervals were randomly distributed, (Gourevitch and Eggermont 2007) for which we report the rate and the fraction of all spikes found in bursts (number of spikes occurring within burst divided by the total number of spikes). Difference of proportion of rhythmic or bursty cell was assessed by *χ*^2^ test. An attempt to isolate the cortical interneurons was performed by selecting spikes with narrow waveforms (McCormick et al. 1985); these data are presented in Supplementary Figure S2. For time–frequency analysis, the average ECoG trace for each animal is going through a Morlet time–frequency transform. The peaks in each of these spectra are searched in the frequency and time intervals to characterize the waves occurring around the stimulation, by taking the norm of the complex time–frequency spectrum and defining the coordinates of the peak of this norm (time, frequency, amplitude of the peak).

## Results

To generate an experimental model of PD, we performed a striatal depletion of dopamine via unilateral injections of the 6-OHDA toxin in the medial forebrain bundle in mice. This procedure yielded an average reduction of the levels of the dopamine synthesis enzyme tyrosine hydroxylase on the lesioned side of 85.5% ± 1.54 (*n* = 39) in lesioned animals versus 11.1% ± 1.88 (*n* = 12) in sham-operated animals (Supplementary Fig. S1). Lesioned animals exhibited a reduced locomotor activity in a circular open field in the 3 weeks following the lesion as revealed by a decrease in the total distance traveled (Fig. 1*A*,*B*, lesion *F*[1,30] = 85.9, *P* < 0.001 week: *F*[2,52] = 12.44, *P* < 0.001, *n* mice 6-OHDA: 20, SHAM: 12, with no difference between the mice before surgeries SHAM 1.62 × 10^3^ ± 125 cm vs. 1.86 × 10^3^ ± 115 cm in 5 min *F*[1,30] = 0.41, *P* = 0.53). This decrease exhibited only a mild recovery over the 3 weeks postlesion (the difference between SHAM and 6-OHDA groups was not dependent on the weeks: interaction Lesion*Week *F*[2,52] = 0.79, *P* = 0.46). This reduction was accompanied with a reduction in the average velocity during the movements bouts (Lesion *F*[1,29] = 27.3, *P* < 0.001; Week: *F*[2,49] = 9.41, *P* < 0.001; Lesion*Week: *F*[2,49] = 1.39, *P* = 0.26. Velocity for the Week 3:6-OHDA: 7.1 ± 0.4 cm/s vs. SHAM: 9.2 ± 0.4 cm/s). This reduced activity was accompanied with a strong increase in the occurrence of contracted body postures (Fig. 1*C*, Lesion: *F*[1,30] = 33.76, *P* < 0.001); these contracted postures are correlated with the increased locomotor immobility (correlation Posture vs. Immobility for 3 weeks: *F*[1,54] = 23.02, *P* < 0.001), but the lesion did not affect differently this dependency (interaction with Lesion: *F*[1,54] = 2.37, *P* = 0.13). The presence of lateralized impairment following unilateral 6-OHDA lesion were assessed in the cylinder test: when rearing against the walls of vertical glass cylinder, the lesioned animals exhibited a decreased usage of the forelimb contralateral to the lesion (Fig. 1*D*, Lesion, *F*[1,19] = 13.55, *P* = 0.002 Week: *F*[2,36] = 0.19, *P* = 0.82, Lesion*Week: *F*[2,36] = 0.956, *P* = 0.394, *n* mice 6-OHDA: 12, SHAM: 9). Overall these observations are consistent with the known effect of unilateral dopaminergic lesions in rodents (Iancu et al. 2005; Glajch et al. 2012; Heuer et al. 2012; Carvalho et al. 2013).

### Cerebellum-Evoked Movements

To test whether the cerebellum exhibits changes in its coupling with the motor effectors, we examined the movements elicited by optogenetic stimulations of Purkinje cells (Chaumont et al. 2013) in the lobule Crus I of the cerebellum in freely moving mice. These stimulations generally generated head rotations, which we measured with an inertial sensor (Dugue et al. 2017) placed on the head of the animal and measuring head rotations with a 5 ms time resolution (Fig. 2*A*). In the basal state, we found that on average, head rotations were slower in lesioned animals (average peak rotational speed—i.e., Euclidian norm of rotational velocity—SHAM: 53.2 ± 2.93 deg/s *n* = 10, 6-OHDA: 34.7 ± 2.15 °/s *n* = 12, *P* < 0.001, Fig. 2*B*) consistent with a lesion-induced bradykinesia. Interestingly, no obvious difference can be noted on the duration of head immobility periods (defined as periods of at least 1 s with head rotational speed under 20 deg/s: SHAM 5.2 ± 0.7 s vs. 6-OHDA 8.0 ± 1.6 s, *P* = 0.28), or on the fraction of time spent in this (head) immobility state (SHAM: 51.5 ± 5.7 % vs. 6-OHDA 59.3 ± 6.6 %, *P* = 0.3), which contrasts with the longer fraction of time with locomotor immobility (no distance traveled) observed in the lesioned mice in openfield (see previous paragraph; ANOVA Lesion: *F*[1,20] = 23.14, *P* < 0.001, Week: *F*[2,39] = 11.5, *P* < 0.001, Lesion * Week: *F*[2,39] = 1.15, *P* = 0,32; for the third week postlesion: SHAM: 50.7 ± 0.03.8 % of time vs. 6-OHDA 77.9 ± 4.1 % of time, *P* = 0.001). This indicates that lesioned animals spend less time traveling in the open field but have normal amounts of head movements, albeit with a smaller speed. Time–spectrum analysis of the head translational acceleration and rotational speed did not reveal any increase in frequency peaks in the low frequency bands related to tremor (except possibly in one lesioned animal which showed brief episodes of oscillations between 10 and 20 Hz not observed in sham-operated animals, Supplementary Fig. S3).

When stimulating Purkinje cells during rest-periods of freely moving mice, we found that mice generated small movements at the onset of the stimulation, and larger head movements shortly after the end of the stimulation (Fig. 2*C*–*K*). The axis of the peak rotations during and after the stimulation varied across trials but were clustered around a direction in most mice (Fig. 2*E*–*G*), although some sham-operated mice showed less directional preference for these rotations as evidenced by the length of the “resultant vector” (see Material and Methods), possibly due to spontaneous movements occurring together with the stimulation-evoked movements. These movements peaked about 200 ms after the stimulation offset and were similar in sham-operated and lesioned animals (peak rotational speed: SHAM: 34.8 ± 9.48 deg/s vs. 6-OHDA: 38.6 ± 10.3 deg/s, *P* = 0.81; latency to peak SHAM: 242 ± 47.4 ms vs. 6-OHDA: 184 ± 33.6 ms, *P* = 0.42, Fig. 2*I*,*K*). Such movements were also observed when stimulating the animals during motor activity, but, in contrast with stimulations performed in the resting state, an increase of head rotations was also observed following the onset of the stimulations, although with a slightly shorter latency to peak than in poststimulation movements (peak rotational speed: SHAM: 24.3 ± 5.55 deg/s vs. 6-OHDA 27.6 ± 6.72 deg/s, *P* = 1; latency to peak: SHAM: 111 ± 27 ms vs. 6-OHDA: 131 ± 40 ms, *P* = 1; Fig. 2*F*,*G*). The rotational speed of movements evoked poststimulation when starting from an active condition were very similar in sham-operated and 6-OHDA-lesioned animals (peak rotational speed: SHAM 24.8 ± 7.2 deg/s vs. 6-OHDA: 31.7 ± 8.4 deg/s, *P* = 1; latency to peak: SHAM: 111 ± 27 ms vs. 6-OHDA: 131 ± 40 ms, *P* = 1). This indicates that the cerebellum-evoked movements, which reflect the combination of cerebellar excitability and coupling of the cerebellum to motor neurons, are not strongly affected in our PD model.

### Motor Cortex and Cerebellum Firing

We then examined the impact of 6-OHDA lesions on the neuronal activity in the corticocerebellar system. For this purpose, we performed recordings in the motor cortex M1 and in the cerebellar nuclei in freely moving mice (Fig. 3). We found that the firing rate of motor cortex M1 of lesioned mice was significantly reduced compared to sham-operated mice (Fig. 3*A*,*B*, *F*[1,12] = 22.8, *P* < 0.001, SHAM 458 cells sampled over 3 weeks from 5 mice. 6-OHDA: 870 cells sampled over 3 weeks from 9 mice; interaction Firing rate * Week: *F*[2 1310] = 6.94, *P* = 0.001; for the third week, the firing rate was mean ± sd SHAM: 0.96 ± 0.88 Hz, 6-OHDA: 0.47 ± 0.39 Hz ANOVA Lesion: *F*[1,12] = 10.8, *P* = 0.007). Only considering cells with broad spikes in motor cortex M1 to remove putative interneurons (McCormick et al. 1985) from the sample did not affect qualitatively this result (Supplementary Fig. S2). In contrast with motor cortex M1, the average firing rate in the cerebellar nuclei was increased following the dopaminergic lesion (Fig. 3*C*,*D*, fastigial nucleus *F*[1,8] = 10, *P* = 0.013; interpositus nucleus *F*[1,9] = 19, *P* = 0.002; dentate nucleus *F*[1,9] = 29, *P* = 0.0005; SHAM: total *n* = 420 cells in 3 weeks, 9 mice, 6-OHDA: total *n* = 574 cells in 3 weeks in 4 mice; firing rate for the week 3: Fastigial SHAM: 9.6 ± 5.0 Hz, 6-OHDA: 19.3 ± 9.2 Hz, Interposed: SHAM: 10.1 ± 5.9 Hz, 6-OHDA: 16.7 ± 8.5, Dentate: SHAM:9.9 ± 4.2 Hz, 6-OHDA: 16.0 ± 6.7 Hz). We also examined whether the cells’ firing patterns were modified by the lesion resulting in an increased presence of bursts (Gourevitch and Eggermont 2007). In both structures motor cortex M1 and cerebellar nuclei, we found that the changes in rate of bursts paralleled the change in firing rate while the fraction of spikes found in burst exhibited only mild changes following the 6-OHDA lesion (Table 1), indicating only limited changes in the firing patterns for the population of neurons sampled. The firing rate irregularity, as measured as the coefficient of variation of the interspikes intervals, failed to show significant difference across group (ANOVA for third week: M1 *F*[1,12] = 0.17, *P* = 0.69, Power=0.067; fastigial nucleus, *F*[1,6] = 2.4, *P* = 0.176, Power = 0.25; interpositus nucleus *F*[1,7] = 0.74, *P* = 0.42, Power = 0.12; dentate nucleus *F*[1,8] = 1.3, *P* = 0.29, Power = 0.17). These results show that the cerebellar nuclei activity is modified in the opposite direction to the primary motor cortex but do not exhibit a strong reorganization of their firing patterns.

**Table 1.** Summary of the bursting characteristics of neurons in motor cortex M1 and the cerebellar nuclei in the third week after the 6-OHDA lesion

Third week postlesion		Rate of bursts			Fraction of spikes in burst			Number of cells
Region	Treatment	Mean ± sem	Repeated-measure ANOVA		Mean ± sem	Repeated-measure ANOVA		
M1	SHAM	0.024 ± 0.001 Hz	*F*(1,12) = 18.8	*P* < 0.001	19.7 ± 0.5 %	*F*(1,12) = 8.71	*P* = 0.012	168
	6-OHDA	0.009 ± 0.001 Hz			14.1 ± 0.5 %			271
Fastigial	SHAM	0.187 ± 0.0273 Hz	*F*(1,6) = 5.8	*P* = 0.052	18.9 ± 1.2 %	*F*(1,6) = 7.2	*P* = 0.04	15
	6-OHDA	0.376 ± 0.025 Hz			14.5 ± 0.4 %			47
Interpositus	SHAM	0.181 ± 0.0228 Hz	*F*(1,7) = 15	*P* = 0.006	16.0 ± 1.0 %	*F*(1,7) = 0.61	*P* = 0.46	23
	6-OHDA	0.33 ± 0.024 Hz			14.4 ± 0.3 %			49
Dentate	SHAM	0.188 ± 0.0105 Hz	*F*(1,8) = 6.1	*P* = 0.039	14.8 ± 0.6 %	*F*(1,8) = 1.2	*P* = 0.30	58
	6-OHDA	0.306 ± 0.0173 Hz			14.8 ± 0.3 %			53

Since the cerebellar nuclei receive excitatory inputs from the motor cortex via pontine nuclei, it could be expected that the changes in firing rate in these structures occur in the same direction. As the cerebellar nuclei also receive strong direct inhibitory inputs from the Purkinje cells, we investigated their changes of firing following dopaminergic lesions. We recorded Purkinje cells in the Crus hemispheres from sham-operated (15 cells from 3 mice) and lesioned (16 cells from 6 mice) mice which express the channelrhodopsin in the Purkinje cells (Chaumont et al. 2013; Figs 4 and 2*C*). Overall, the cells in the 6-OHDA-lesioned animals exhibited only mild, nonsignificant, changes in the population average of the firing rate and discharge irregularity compared to sham-operated animals (rate SHAM: 43.1 ± 7.41 Hz vs. 6-OHDA: 27.4 ± 8.48 Hz, ANOVA: *F*[1,7] = 3.85, *P* = 0.09 power=0.40; coefficient of variation of interspike intervals SHAM: 0.52 ± 0.09 vs. 6-OHDA: 1.49 ± 0.51, *P* = 0.049, power=0.16). We found little change in the occurrence of firing bursts (SHAM: 0.62 ± 0.11 Hz vs. 6-OHDA: 0.45 ± 0.2 Hz, *P* = 0.054), and in the fraction of spikes in burst (SHAM 13.3 ± 0.1% vs. 6-OHDA 15.0 ± 2.6 %, *P* = 0.06). However, the distribution of Purkinje cell firing rate was altered by the dopaminergic lesion (Fig. 4*B*, inset, *P* = 0.019, Kolmogorov–Smirnov test), with notably a large fraction of cells in 6-OHDA-lesioned mice exhibiting a low firing rate (8/16, i.e., 50% of cells with an average firing rate under 10 Hz in lesioned animals vs. 1/15 in sham-operated animals; $\chi^{2}$ = 7.06, *P* = 0.008). The firing of these slow cells was characterized either as slow continuous firing, or with only complex spike firing remaining (Fig. 4*A*, bottom). We found a combination of normal and slow Purkinje cells in 2 lesioned animals, suggesting that these populations coexist after dopaminergic degeneration. Overall, these results indicate that the firing of a subset of Purkinje cells is reduced, and may thus impose a lower inhibition to cerebellar nuclei cells and favor their higher firing rates as noted above (Fig. 3).

### Coupling of the Cerebellum and Motor Cortex

**Figure 5.**
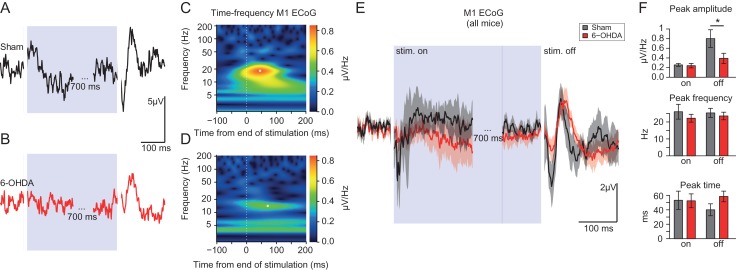
Electrocorticogram (ECoG) response in the primary motor cortex to cerebellar stimulations. (*A*, *B*) Example average ECoG response to cerebellar stimulations in a sham-operated (*A*) and a 6-OHDA-lesioned animal. Note the response evoked at the end of the cerebellar stimulations (*B*). (*C*, *D*) Time–frequency analysis of the poststimulation ECoG responses displayed in *A*, *B*. The ECoG responses appear as peak in the 15–40 Hz range in the 100 ms following the end of the optogenetic cerebellar stimulation. (*E*) Average traces from 10 sham-operated (black) and 12 6-OHDA-lesioned (red) mice; note the multiphasic response at the end of the stimulations. Traces are mean ± sem. (*F*) Quantification of time–frequency peak coefficient (amplitude), frequency (width) and latency, in the 15–10 ms and 15–40 Hz range for the ECoG traces after the stim onset (“on,” and after the end of the stim (“off”); for “on” see values in text. For “off,” there was no significant difference although the power was small (respectively Student’s *t*-test: *P* = 0.43, 0.47, and 0.94, and the power 0.05, 0.12, 0.06 for amplitude, frequency, time of the peak).

To examine directly the coupling of the cerebellar cortex to the cerebral cortex, we performed Purkinje cells optogenetic stimulations on the cerebellar hemisphere and recorded the electrical potential on the surface of the contralateral motor cortex (Fig. 5). Changes of cortical surface potential measured by the Electrocorticogram (ECoG) were small or absent at the onset of cerebellar optogenetic stimulations, while consistent ECoG responses were observed at the end of 1 s stimulation in the form of a multiphasic wave (Fig 5*A*,*B*,*E*). These poststimulation responses are similar to the postinhibitory rebound excitation of the cerebellar nuclei following 100 ms stimulation of the cerebellar cortex, which were shown to propagate via the thalamus up to the cerebral cortex (Proville et al. 2014). The average of the evoked potential waves following the end of the stimulation was smaller in lesioned than in sham-operated animals (Fig. 5*E*). These observations were confirmed when the ECoG was averaged only for stimulations occurring while the animal was only resting or only active (Supplementary Fig. S4A–F). To quantify the position and amplitude of these waves for each animal, we designed a Morlet time–frequency approach; indeed, the poststimulations waves appeared as peaks in the range of 15–40 Hz in time–frequency plots (Fig. 5*C*,*D*), where the time of the peak correspond to the time of the largest oscillation, and the inverse of the peak frequency reflects the width of the wave. We found that the wave occurring following cerebellar stimulations was smaller in the 6-OHDA-lesioned compared to sham-operated animals (Fig. 5*F*, coefficient, i.e., response size at peak: SHAM: 0.80 ± 0.18 μV vs. 6-OHDA: 0.39 ± 0.11 μV, *P* = 0.04; time of peak: SHAM 40 ± 8 ms vs. 6-OHDA 58 ± 7 ms, *P* = 0.08, power = 0.11; frequency (reflecting response width) at peak: SHAM 25 ± 3 Hz vs. 6-OHDA 23 ± 2 Hz *P* = 0.69, power = 0.62; *n* mice: SHAM 10, 6-OHDA 12; no difference was found for the response at stimulation onset, see legend of Fig. 5). For lower frequencies (corresponding to waves larger than 100 ms), we found no difference in amplitude of time–frequency peaks but a trend to occur earlier in lesioned animals (Supplementary Fig. S4G); a difference in the long-latency ECoG responses to cerebellar stimulations due to weakening to polysynaptic responses in lesioned animals could be present, albeit difficult to establish with our dataset. Overall, these results indicate a weaker response of the cerebral cortex in 6-OHDA-lesioned animals following cerebellar optogenetic stimulation.

## Discussion

In the current study, we investigated the functional coupling of cerebellum and motor cortex in a mouse model for Parkinson disease (PD). We confirmed the strong impact of dopaminergic lesion on locomotion, posture and limb use. In contrast, optogenetic stimulations of Purkinje cells in the cerebellar cortex generated head movements, which exhibited normal latency and amplitude after dopaminergic lesion consistent with a normal coupling of the cerebellum with motor effectors in our PD model. While a reduced firing was observed in the motor cortex M1, 6-OHDA-lesioned animals exhibited an increased firing rate in the deep cerebellar nuclei. Moreover, we found a robust reduction in firing rate from a subset of Purkinje cells in 6-OHDA-lesioned animals, suggesting that the increased firing rates of cerebellar nuclei neurons resulted in part from a reduced inhibition from the cerebellar cortex. However, cerebellar optogenetic stimulations revealed a weakened cerebello–cerebral coupling, which may thus reduce the ability of cerebellar nuclei over-activation to directly compensate for motor cortex M1 depression by providing increased excitatory cerebello–thalamo–cortical inputs.

### Cerebellar Coupling to Movement and Motor Cortex in Parkinson Disease

In control and 6-OHDA-lesioned mice, optogenetic stimulations of hemispheric Purkinje cells in the cerebellum induced head movement at the offset of the stimulations. The optogenetic activation of Purkinje cells at rest has been shown to induce a fast and rapidly reversible inhibition of the nuclear neurons (Chaumont et al. 2013) with movement evoked not during the stimulation but after the end of the stimulation, at the time when nuclear neurons restart firing (Proville et al. 2014; Lee et al. 2015); the timing at which the wave is triggered after Purkinje cell activation depends on the intensity of the inhibition imposed on nuclear neurons (see Fig. 3*E* in Chaumont et al. 2013). Indeed, disinhibition of deep cerebellar nuclei neurons potently triggers movements with short latency (Heiney et al. 2014; Lee et al. 2015).

Optogenetic stimulations of the Purkinje cells may modify ongoing movements (Proville et al. 2014; Sarnaik and Raman 2018). Our unilateral stimulations targeted to Purkinje cells in the Crus I lobule produced discrete effects on the behavior of the mice, mostly limited to head movements. When recorded from rest, the movements following optogenetic stimulation onset were small and slow (<10 deg/s) and typically contraversive, consistent with an inhibition of cerebellar nuclei neurons controlling neck muscle contractions ipsilateral to the stimulation side. Larger movements occurred at the end of the stimulation consistent with a rebound effect in the cerebellar nuclei (see above). The profile of head rotational speed for movements evoked at the end of the stimulation from rest or during movements was similar in sham-operated and 6-OHDA-lesioned animals. Examination of the orientation of the rotations however indicated mild differences between the groups; while all lesioned animals exhibited ipsiversive head rotations, head rotations in sham-operated were less consistent (in 3 sham-operated mice, the axis of the evoked rotations was very variable, and in 3 other mice, the rotation was not ipsiversive). This suggests that the strength of the entrainment of neck muscles by the cerebellum and of the modulatory action of cerebellar cortex on these muscles are little affected by the dopaminergic lesion, but that the repertoire of evoked movements is smaller after the lesion.

How cerebellar stimulations over Crus I elicit head movements remains uncertain. Considering that microstimulation of vermal lobule 6, which would correspond to a more medial area than the one targeted in our study, triggers neck muscle activation in humans (Mottolese et al. 2013) and that the same area receives sensory inputs in cats (Berthoz and Llinas 1974), these vermal areas likely contribute to the postural control and orientation system via the fastigial nucleus (Pelisson et al. 1998). Neck-related sensory activities are also found in the hemisphere (lobule V pars intermedia, Berthoz and Llinas 1974) and interpositus nucleus (Boyle and Pompeiano 1980) suggesting that cerebellar hemispheres also contribute to head movements. Indeed, lateral nucleus stimulation elicit movements even following cerebral cortex lesion (Cicirata et al. 1989), consistent with a limited role of ascending cerebello–cortical projections in eliciting movements (Asanuma and Hunsperger 1975). Therefore, the similarity of head movements evoked by cerebellar stimulations in 6-OHDA-lesioned and sham-operated animals in the present study are consistent with a normal coupling of cerebellum to motor neck effectors via projections to the red nucleus or reticular formation following dopaminergic lesion, and contrasts with a decreased response of motor cortex, which is mediated via the diencephalic areas (Proville et al. 2014), in lesioned animals.

The motor cortex M1 ECoG response to Purkinje cell stimulation is very similar to the one studied in the Figures 4 and 5 and Suppl. Fig. S6 of Proville et al. (2014), and results from the strong rebound activation of the cerebello–thalamo–cortical axis after the inhibition of cerebellar nuclei by the stimulated Purkinje cells; the same study showed that during the stimulation, the inhibition of cerebellar nuclei was only associated with a mild reduction of motor cortex M1 firing, which produces as a small or no ECoG response. The reduction in motor cortex M1 ECoG response to cerebellar stimulation suggests the presence of pathway-specific adaptations in the cerebello–cerebral pathways following dopaminergic lesion. Such disruption has also been observed in humans using cerebellar cortex transcranial stimulations modulating motor evoked potentials (Ugawa et al. 1991), an effect called “cerebellar brain inhibition.” In healthy subjects the stimulation of the cerebellar cortex causes a transient (<10 ms) reduction of the electromyogram potentials evoked by transcranial magnetic stimulations of the motor cortex. The interpretation of this effect is that transcranial stimulation of the cerebellum excites the Purkinje cells and consequently inhibits the excitatory cerebello–thalamo–cortical pathway, leading to a reduced excitability of the motor cortex. In PD patients, recent evidence indicates that this inhibitory effect of cerebellum on motor cortex is lost (or even converted to a slight excitation) when patients are off-treatment (Molnar et al. 2004; Ni et al. 2010; Carrillo et al. 2013); studies under levodopa treatment or deep-brain stimulation led to more divergent observations (Molnar et al. 2004; Shirota et al. 2010; Carrillo et al. 2013). Alterations of cerebellar control over the motor cortex in PD patients were reported for the recruitment of short-term intracortical inhibition (Carrillo et al. 2013), the modulation of LTP-like associative sensory-motor plasticity (Kishore et al. 2014) and the interaction between cerebello–thalamo–cortical and cortical afferents (Lu et al. 2017). The link between these anomalies and the involvement of the cerebellum in PD tremor (Timmermann et al. 2003; Ni et al. 2010; Helmich et al. 2012) is yet unclear; the disruption of cerebellar brain inhibition has been correlated with tremor-resetting effect of cerebellar stimulations consistent with a contribution of the cerebello–thalamo–cortical pathway to tremor generation (Molnar et al. 2004; Ni et al. 2010). The lack of obvious tremor in our accelerometric measures suggests that disruption of the cerebello–cortical functional connectivity is not exclusively related with tremor (Lewis et al. 2011) and may thus rather reflect a primary abnormality in PD (Ni et al. 2010).

The depression of the cerebello–cortical pathway shall deprive the cortical neurons from the cerebellar contribution to their firing pattern; indeed, in experimental PD, pyramidal tract neurons fail to exhibit a strong and temporal tuning to the movement (Pasquereau et al. 2016), a feature found in cortical neurons receiving short-latency inputs from the cerebellum (Nashef et al. 2018) and perturbed or suppressed by cooling of the cerebellar nuclei (Meyer-Lohmann et al. 1977; Hore and Flament 1988). Therefore, the deficits in firing of motor cortex M1 pyramidal tract neurons could be in part consecutive to the depressed cerebello–cortical coupling.

### Changes in Firing Rates

To explore physiological alterations in the cerebellum after dopaminergic depletion beyond the functional connectivity discussed above, we recorded single unit activity in the cerebellum and motor cortex M1. In 6-OHDA-lesioned animals, we observed a decrease in firing rate, as well as a mild change of bursting pattern, in the spontaneous activity of the neurons in the motor cortex M1 ipsilateral to the lesion. The reduction in firing rate was similar to that reported in unanaesthetized primates and rodent PD models (Pasquereau and Turner 2011; Li et al. 2012). This decreased activity in the motor cortex M1 is usually explained by an increased inhibition of thalamo–cortical neurons by the basal ganglia (for review see Valls-Sole and Valldeoriola 2002; DeLong and Wichmann 2007). This result is also in line with the decreased cortical excitability reported in 6-OHDA-lesioned rodents (Viaro et al. 2011) and the correlation between such decrease and bradykinesia found in PD patients (Ellaway et al. 1995). We did not find prominent increase in cortical bursting activity, it remains possible that this might occur differently across population of cortical neurons (Ballion et al. 2008; Pasquereau and Turner 2011; Degos et al. 2013).

Compared to control, we found an increase in the firing rate of deep cerebellar neurons in 6-OHDA-lesioned animals. Our average cerebellar nuclei firing rate was lower than in other reports in vivo (Fremont et al. 2017; White and Sillitoe 2017; Sarnaik and Raman 2018); the reason for this is unclear but may result from a bias of our electrodes sensitivity toward small cells, which have been reported to exhibit slower firing rates in vivo (Canto et al. 2016). The increased firing rate in cerebellar nuclei contrasts with the decreased firing rate in M1. Since the cortex reaches all cerebellar nuclei by way of the pontine nuclei (Mihailoff 1993), the increased cerebellar nuclei activity unlikely reflects changes in cortical inputs. Instead, our recordings of Purkinje cells, which provide inhibitory inputs to the nuclei, suggest that these changes could reflect decreased inhibition. We identified a fraction of Purkinje cell exhibiting an unusual very low and irregular firing pattern. Purkinje cells can produce spontaneously simple spikes in a tonic pattern usually described around 50–80 Hz (Cheron et al. 2014) but this tonic state may be interrupted by periods of silence (Loewenstein et al. 2005). These transitions are caused by shifts of membrane potential between hyperpolarized and depolarized states, respectively called down- and up-states (Engbers et al. 2013). Down-states are more frequently observed in anaesthetized animals (Schonewille et al. 2006) but have also been reported in awake animals (Yartsev et al. 2009). In 6-OHDA-lesioned mice, some Purkinje cells exhibited silence periods indicative of down states but also low firing rate of Purkinje cells during phases of tonic activity. Interestingly, a fraction of Purkinje cells in 6-OHDA-lesioned animals exhibited normal behavior, suggesting that only specific sets of neurons are affected. An attractive hypothesis is that these cells would be more specifically engaged in a cerebrocerebellar circuit (Léna and Popa 2015). Changes in the pattern and rate of Purkinje cell firing has also been reported in other motor disorders, such as dystonia (Sausbier et al. 2004; Fremont et al. 2014, 2015), ataxia (Hansen et al. 2013; Egorova et al. 2016; Jayabal et al. 2016), and autism spectrum disorders (Tsai et al. 2012; Cupolillo et al. 2016). Abnormal firing patterns in models of dystonia are associated with increased c-Fos expression, and indeed, abnormal c-Fos expression has been also reported in Purkinje cells following MPTP lesions of dopaminergic neurons (Necchi et al. 2004; Heman et al. 2012). In the long term the changes in Purkinje cell activity could be causing increased death of these cells (Necchi et al. 2004).

### Clinical Implications

The thalamic relay in the cerebello–thalamo–cortical pathway is a therapeutic target for the deep brain stimulations in the treatment of PD tremor (Wu and Hallett 2013) and such stimulations may indeed improve the cerebello–thalamo–cortical functional connectivity (Molnar et al. 2004). The subthalamic nucleus is also an effective target for the treatment of PD conditions and the beneficial effect of subthalamic stimulations could be exerted via antidromic recruitment of cortical afferents (Gradinaru et al. 2009). Interestingly, the subthalamic nucleus sends also disynaptic projections to the cerebellar cortex (Bostan and Strick 2010). Glutamatergic projections could be relayed by the pontine nuclei, although few neurons connect the 2 structures (Giolli et al. 2001). Alternatively, recent anatomical evidence rather points toward a cholinergic relay in the pedunculo-pontine area (Jwair et al. 2017), particularly with the dentate nucleus in the cerebellum (Vitale et al. 2016). Therefore, the high-frequency stimulation of the subthalamic nucleus may indeed engage the cerebellum, decreasing spiking activity in Purkinje cells and increasing deep cerebellar nuclei spiking activity (Sutton et al. 2015) and c-Fos expression (Moers-Hornikx et al. 2011) raising the possibility that STN stimulations could directly impact cerebellar function in PD. In the recent years, several procedures of transcranial stimulation targeted at the cerebellum have been developed and tested in patients (Franca et al. 2018). These procedures have so far limited efficacy in PD except for the treatment of levodopa-induced dyskinesia (Koch et al. 2009; Kishore et al. 2014). However, our data in Purkinje cells suggest that only a fraction of the cerebellar circuits is malfunctioning, and the methods employed so far to modify cerebellar function in patients may be too coarse to target subsets of cells. Moreover, the recent demonstration that the cerebello–thalamo–striatal pathway provides a powerful excitation of the striatal neurons (Chen et al. 2014) suggests that the cerebellum could also provide a therapeutic gateway to the striatum.

## Conclusions

Our study shows that the meso-striatal dopaminergic lesion leads to differential effects of cerebellar output pathways on movement and motor cortex. Increases of firing rate in the cerebellum could promote an increased excitatory drive to its targets. However, the compensatory benefits of these changes on the low motor cortex M1 firing rate are likely hampered by the functional uncoupling between the cerebellum and the motor cortex. The normal coupling of the cerebellum to descending pathways may explain the lack of classical “cerebellar” motor symptoms in PD patients, while the specific disruption of the ascending cerebellar output pathway may explain the weaker amplitude and temporal-tuning of cortical neuronal firing to movement, which requires cerebellar inputs. Identifying strategies to improve cerebello–cortical coupling in PD could therefore help design new therapeutic strategies.

## Supplementary Material

Supplementary Data

## References

[bhy346C1] Albin RL, Young AB, Penney JB 1989 The functional anatomy of basal ganglia disorders. Trends Neurosci. 12:366–375. 2479133 10.1016/0166-2236(89)90074-x

[bhy346C2] Alexander GE, DeLong MR, Strick PL 1986 Parallel organization of functionally segregated circuits linking basal ganglia and cortex. Annu Rev Neurosci. 9:357–381. 3085570 10.1146/annurev.ne.09.030186.002041

[bhy346C3] Asanuma H, Hunsperger RW 1975 Functional significance of projection from the cerebellar nuclei to the motor cortex in the cat. Brain Res. 98:73–92. 1175060 10.1016/0006-8993(75)90510-7

[bhy346C4] Ballion B, Mallet N, Bezard E, Lanciego JL, Gonon F 2008 Intratelencephalic corticostriatal neurons equally excite striatonigral and striatopallidal neurons and their discharge activity is selectively reduced in experimental parkinsonism. Eur J Neurosci. 27:2313–2321. 18445222 10.1111/j.1460-9568.2008.06192.x

[bhy346C5] Benabid AL, Pollak P, Gervason C, Hoffmann D, Gao DM, Hommel M, Perret JE, de Rougemont J 1991 Long-term suppression of tremor by chronic stimulation of the ventral intermediate thalamic nucleus. Lancet. 337:403–406. 1671433 10.1016/0140-6736(91)91175-t

[bhy346C6] Bergman H, Wichmann T, Karmon B, DeLong MR 1994 The primate subthalamic nucleus. II. Neuronal activity in the MPTP model of parkinsonism. J Neurophysiol. 72:507–520. 7983515 10.1152/jn.1994.72.2.507

[bhy346C7] Berthoz A, Llinas R 1974 Afferent neck projection to the cat cerebellar cortex. Exp Brain Res. 20:385–401. 4426359 10.1007/BF00237383

[bhy346C8] Bezard E, Gross CE, Brotchie JM 2003 Presymptomatic compensation in Parkinson’s disease is not dopamine-mediated. Trends Neurosci. 26:215–221. 12689773 10.1016/S0166-2236(03)00038-9

[bhy346C9] Blandini F, Nappi G, Tassorelli C, Martignoni E 2000 Functional changes of the basal ganglia circuitry in Parkinson’s disease. Prog Neurobiol. 62:63–88. 10821982 10.1016/s0301-0082(99)00067-2

[bhy346C10] Bostan AC, Strick PL 2010 The cerebellum and basal ganglia are interconnected. Neuropsychol Rev. 20:261–270. 20811947 10.1007/s11065-010-9143-9 PMC3325093

[bhy346C11] Boyle R, Pompeiano O 1980 Response characteristics of cerebellar interpositus and intermediate cortex neurons to sinusoidal stimulation of neck and labyrinth receptors. Neuroscience. 5:357–372. 7374947 10.1016/0306-4522(80)90111-6

[bhy346C12] Canto CB, Witter L, De Zeeuw CI 2016 Whole-cell properties of cerebellar nuclei neurons in vivo. PLoS One. 11:e0165887. 27851801 10.1371/journal.pone.0165887 PMC5112928

[bhy346C13] Carrillo F, Palomar FJ, Conde V, Diaz-Corrales FJ, Porcacchia P, Fernandez-Del-Olmo M, Koch G, Mir P 2013 Study of cerebello-thalamocortical pathway by transcranial magnetic stimulation in Parkinson’s disease. Brain Stimul. 6:582–589. 23318222 10.1016/j.brs.2012.12.004

[bhy346C14] Carvalho MM, Campos FL, Coimbra B, Pego JM, Rodrigues C, Lima R, Rodrigues AJ, Sousa N, Salgado AJ 2013 Behavioral characterization of the 6-hydroxidopamine model of Parkinson’s disease and pharmacological rescuing of non-motor deficits. Mol Neurodegener. 8:14. 23621954 10.1186/1750-1326-8-14 PMC3653696

[bhy346C15] Chaumont J, Guyon N, Valera AM, Dugue GP, Popa D, Marcaggi P, Gautheron V, Reibel-Foisset S, Dieudonne S, Stephan A, et al 2013 Clusters of cerebellar Purkinje cells control their afferent climbing fiber discharge. Proc Natl Acad Sci USA. 110:16223–16228. 24046366 10.1073/pnas.1302310110 PMC3791757

[bhy346C16] Chen CH, Fremont R, Arteaga-Bracho EE, Khodakhah K 2014 Short latency cerebellar modulation of the basal ganglia. Nat Neurosci. 17:1767–1775. 25402853 10.1038/nn.3868 PMC4241171

[bhy346C17] Cheron G, Prigogine C, Cheron J, Marquez-Ruiz J, Traub RD, Dan B 2014 Emergence of a 600-Hz buzz UP state Purkinje cell firing in alert mice. Neuroscience. 263:15–26. 24440752 10.1016/j.neuroscience.2014.01.007 PMC4860878

[bhy346C18] Cicirata F, Angaut P, Panto MR, Serapide MF 1989 Neocerebellar control of the motor activity: experimental analysis in the rat. Comparative aspects. Brain Res Brain Res Rev. 14:117–141. 2752228 10.1016/0165-0173(89)90011-8

[bhy346C19] Cupolillo D, Hoxha E, Faralli A, De Luca A, Rossi F, Tempia F, Carulli D 2016 Autistic-like traits and cerebellar dysfunction in purkinje cell PTEN knock-out mice. Neuropsychopharmacology. 41:1457–1466. 26538449 10.1038/npp.2015.339 PMC4832032

[bhy346C20] Degos B, Deniau JM, Chavez M, Maurice N 2009 Chronic but not acute dopaminergic transmission interruption promotes a progressive increase in cortical beta frequency synchronization: relationships to vigilance state and akinesia. Cereb Cortex. 19:1616–1630. 18996909 10.1093/cercor/bhn199

[bhy346C21] Degos B, Deniau JM, Chavez M, Maurice N 2013 Subthalamic nucleus high-frequency stimulation restores altered electrophysiological properties of cortical neurons in parkinsonian rat. PLoS One. 8:e83608. 24391793 10.1371/journal.pone.0083608 PMC3877054

[bhy346C22] DeLong MR, Wichmann T 2007 Circuits and circuit disorders of the basal ganglia. Arch Neurol. 64:20–24. 17210805 10.1001/archneur.64.1.20

[bhy346C23] Dirkx MF, den Ouden HE, Aarts E, Timmer MH, Bloem BR, Toni I, Helmich RC 2017 Dopamine controls Parkinson’s tremor by inhibiting the cerebellar thalamus. Brain. 140:721–734. 28073788 10.1093/brain/aww331

[bhy346C24] Dugue GP, Tihy M, Gourevitch B, Lena C 2017 Cerebellar re-encoding of self-generated head movements. ELife. 6. pii: e26179. 10.7554/eLife.26179 PMC5489315 28608779

[bhy346C25] Egorova PA, Zakharova OA, Vlasova OL, Bezprozvanny IB 2016 In vivo analysis of cerebellar Purkinje cell activity in SCA2 transgenic mouse model. J Neurophysiol. 115:2840–2851. 26984424 10.1152/jn.00913.2015 PMC4922606

[bhy346C26] Eidelberg D 2009 Metabolic brain networks in neurodegenerative disorders: a functional imaging approach. Trends Neurosci. 32:548–557. 19765835 10.1016/j.tins.2009.06.003 PMC2782537

[bhy346C27] Eidelberg D, Moeller JR, Dhawan V, Spetsieris P, Takikawa S, Ishikawa T, Chaly T, Robeson W, Margouleff D, Przedborski S, et al 1994 The metabolic topography of parkinsonism. J Cereb Blood Flow Metab. 14:783–801. 8063874 10.1038/jcbfm.1994.99

[bhy346C28] Ellaway PH, Davey NJ, Maskill DW, Dick JP 1995 The relation between bradykinesia and excitability of the motor cortex assessed using transcranial magnetic stimulation in normal and parkinsonian subjects. Electroencephalogr Clin Neurophysiol. 97:169–178. 7607106 10.1016/0924-980x(94)00336-6

[bhy346C29] Engbers JD, Fernandez FR, Turner RW 2013 Bistability in Purkinje neurons: ups and downs in cerebellar research. Neural Netw. 47:18–31. 23041207 10.1016/j.neunet.2012.09.006

[bhy346C30] Franca C, de Andrade DC, Teixeira MJ, Galhardoni R, Silva V, Barbosa ER, Cury RG 2018 Effects of cerebellar neuromodulation in movement disorders: a systematic review. Brain Stimul. 11:249–260. 29191439 10.1016/j.brs.2017.11.015

[bhy346C31] Francardo V, Recchia A, Popovic N, Andersson D, Nissbrandt H, Cenci MA 2011 Impact of the lesion procedure on the profiles of motor impairment and molecular responsiveness to L-DOPA in the 6-hydroxydopamine mouse model of Parkinson’s disease. Neurobiol Dis. 42(3):327–340. 21310234 10.1016/j.nbd.2011.01.024

[bhy346C32] Fremont R, Calderon DP, Maleki S, Khodakhah K 2014 Abnormal high-frequency burst firing of cerebellar neurons in rapid-onset dystonia-parkinsonism. J Neurosci. 34:11723–11732. 25164667 10.1523/JNEUROSCI.1409-14.2014 PMC4145175

[bhy346C33] Fremont R, Tewari A, Angueyra C, Khodakhah K 2017 A role for cerebellum in the hereditary dystonia DYT1. ELife. 6. pii: e22775. 10.7554/eLife.22775 PMC5340526 28198698

[bhy346C34] Fremont R, Tewari A, Khodakhah K 2015 Aberrant Purkinje cell activity is the cause of dystonia in a shRNA-based mouse model of Rapid Onset Dystonia-Parkinsonism. Neurobiol Dis. 82:200–212. 26093171 10.1016/j.nbd.2015.06.004 PMC4641034

[bhy346C35] Gale JT, Amirnovin R, Williams ZM, Flaherty AW, Eskandar EN 2008 From symphony to cacophony: pathophysiology of the human basal ganglia in Parkinson disease. Neurosci Biobehav Rev. 32:378–387. 17466375 10.1016/j.neubiorev.2006.11.005

[bhy346C36] Giolli RA, Gregory KM, Suzuki DA, Blanks RH, Lui F, Betelak KF 2001 Cortical and subcortical afferents to the nucleus reticularis tegmenti pontis and basal pontine nuclei in the macaque monkey. Vis Neurosci. 18:725–740. 11925008 10.1017/s0952523801185068

[bhy346C37] Glajch KE, Fleming SM, Surmeier DJ, Osten P 2012 Sensorimotor assessment of the unilateral 6-hydroxydopamine mouse model of Parkinson’s disease. Behav Brain Res. 230:309–316. 22178078 10.1016/j.bbr.2011.12.007 PMC3324279

[bhy346C38] Gourevitch B, Eggermont JJ 2007 A nonparametric approach for detection of bursts in spike trains. J Neurosci Methods. 160:349–358. 17070926 10.1016/j.jneumeth.2006.09.024

[bhy346C39] Gradinaru V, Mogri M, Thompson KR, Henderson JM, Deisseroth K 2009 Optical deconstruction of parkinsonian neural circuitry. Science. 324:354–359. 19299587 10.1126/science.1167093 PMC6744370

[bhy346C40] Hacker CD, Perlmutter JS, Criswell SR, Ances BM, Snyder AZ 2012 Resting state functional connectivity of the striatum in Parkinson’s disease. Brain. 135:3699–3711. 23195207 10.1093/brain/aws281 PMC3525055

[bhy346C41] Hansen ST, Meera P, Otis TS, Pulst SM 2013 Changes in Purkinje cell firing and gene expression precede behavioral pathology in a mouse model of SCA2. Hum Mol Genet. 22:271–283. 23087021 10.1093/hmg/dds427 PMC3526159

[bhy346C42] Heiney SA, Kim J, Augustine GJ, Medina JF 2014 Precise control of movement kinematics by optogenetic inhibition of Purkinje cell activity. J Neurosci. 34:2321–2330. 24501371 10.1523/JNEUROSCI.4547-13.2014 PMC3913874

[bhy346C43] Helmich RC, Hallett M, Deuschl G, Toni I, Bloem BR 2012 Cerebral causes and consequences of Parkinsonian resting tremor: a tale of two circuits? Brain. 135:3206–3226. 22382359 10.1093/brain/aws023 PMC3501966

[bhy346C44] Helmich RC, Janssen MJ, Oyen WJ, Bloem BR, Toni I 2011 Pallidal dysfunction drives a cerebellothalamic circuit into Parkinson tremor. Ann Neurol. 69:269–281. 21387372 10.1002/ana.22361

[bhy346C45] Heman P, Barcia C, Gomez A, Ros CM, Ros-Bernal F, Yuste JE, de Pablos V, Fernandez-Villalba E, Toledo-Cardenas MR, Herrero MT 2012 Nigral degeneration correlates with persistent activation of cerebellar Purkinje cells in MPTP-treated monkeys. Histol Histopathol. 27:89–94. 22127600 10.14670/HH-27.89

[bhy346C46] Heuer A, Smith GA, Lelos MJ, Lane EL, Dunnett SB 2012 Unilateral nigrostriatal 6-hydroxydopamine lesions in mice I: motor impairments identify extent of dopamine depletion at three different lesion sites. Behav Brain Res. 228:30–43. 22146593 10.1016/j.bbr.2011.11.027

[bhy346C47] Hoover JE, Strick PL 1999 The organization of cerebellar and basal ganglia outputs to primary motor cortex as revealed by retrograde transneuronal transport of herpes simplex virus type 1. J Neurosci. 19:1446–1463. 9952421 10.1523/JNEUROSCI.19-04-01446.1999 PMC6786031

[bhy346C48] Hore J, Flament D 1988 Changes in motor cortex neural discharge associated with the development of cerebellar limb ataxia. J Neurophysiol. 60:1285–1302. 3193158 10.1152/jn.1988.60.4.1285

[bhy346C49] Howarth C, Peppiatt-Wildman CM, Attwell D 2010 The energy use associated with neural computation in the cerebellum. J Cereb Blood Flow Metab. 30:403–414. 19888288 10.1038/jcbfm.2009.231 PMC2859342

[bhy346C50] Huang C, Tang C, Feigin A, Lesser M, Ma Y, Pourfar M, Dhawan V, Eidelberg D 2007 Changes in network activity with the progression of Parkinson’s disease. Brain. 130:1834–1846. 17470495 10.1093/brain/awm086 PMC4454378

[bhy346C51] Iancu R, Mohapel P, Brundin P, Paul G 2005 Behavioral characterization of a unilateral 6-OHDA-lesion model of Parkinson’s disease in mice. Behav Brain Res. 162:1–10. 15922062 10.1016/j.bbr.2005.02.023

[bhy346C52] Jayabal S, Chang HH, Cullen KE, Watt AJ 2016 4-aminopyridine reverses ataxia and cerebellar firing deficiency in a mouse model of spinocerebellar ataxia type 6. Sci Rep. 6:29489. 27381005 10.1038/srep29489 PMC4933933

[bhy346C53] Jwair S, Coulon P, Ruigrok TJ 2017 Disynaptic subthalamic input to the posterior cerebellum in rat. Front Neuroanat. 11:13. 28293179 10.3389/fnana.2017.00013 PMC5329055

[bhy346C55] Kishore A, Popa T, Balachandran A, Chandran S, Pradeep S, Backer F, Krishnan S, Meunier S 2014 Cerebellar sensory processing alterations impact motor cortical plasticity in Parkinson’s disease: clues from dyskinetic patients. Cereb Cortex. 24:2055–2067. 23535177 10.1093/cercor/bht058

[bhy346C56] Koch G, Brusa L, Carrillo F, Lo Gerfo E, Torriero S, Oliveri M, Mir P, Caltagirone C, Stanzione P 2009 Cerebellar magnetic stimulation decreases levodopa-induced dyskinesias in Parkinson disease. Neurology. 73:113–119. 19597133 10.1212/WNL.0b013e3181ad5387

[bhy346C57] Lee KH, Mathews PJ, Reeves AM, Choe KY, Jami SA, Serrano RE, Otis TS 2015 Circuit mechanisms underlying motor memory formation in the cerebellum. Neuron. 86:529–540. 25843404 10.1016/j.neuron.2015.03.010 PMC4417109

[bhy346C58] Lees AJ, Hardy J, Revesz T 2009 Parkinson’s disease. Lancet. 373:2055–2066. 19524782 10.1016/S0140-6736(09)60492-X

[bhy346C59] Léna C, Popa D 2015 Cerebrocerebellar loops in the rodent brain In: Heck D, editor The neuronal codes of the cerebellum. Cambridge, Massachusetts, États-Unis: Academic Press-Elsevier p. 135–153.

[bhy346C60] Lewis MM, Du G, Sen S, Kawaguchi A, Truong Y, Lee S, Mailman RB, Huang X 2011 Differential involvement of striato- and cerebello-thalamo-cortical pathways in tremor- and akinetic/rigid-predominant Parkinson’s disease. Neuroscience. 177:230–239. 21211551 10.1016/j.neuroscience.2010.12.060 PMC3049982

[bhy346C61] Li Q, Ke Y, Chan DC, Qian ZM, Yung KK, Ko H, Arbuthnott GW, Yung WH 2012 Therapeutic deep brain stimulation in Parkinsonian rats directly influences motor cortex. Neuron. 76:1030–1041. 23217750 10.1016/j.neuron.2012.09.032

[bhy346C62] Lin TP, Carbon M, Tang C, Mogilner AY, Sterio D, Beric A, Dhawan V, Eidelberg D 2008 Metabolic correlates of subthalamic nucleus activity in Parkinson’s disease. Brain. 131:1373–1380. 18400841 10.1093/brain/awn031

[bhy346C63] Loewenstein Y, Mahon S, Chadderton P, Kitamura K, Sompolinsky H, Yarom Y, Hausser M 2005 Bistability of cerebellar Purkinje cells modulated by sensory stimulation. Nat Neurosci. 8:202–211. 15665875 10.1038/nn1393

[bhy346C64] Lu MK, Chen JC, Chen CM, Duann JR, Ziemann U, Tsai CH 2017 Impaired cerebellum to primary motor cortex associative plasticity in Parkinson’s disease and spinocerebellar ataxia type 3. Front Neurol. 8:445. 28900413 10.3389/fneur.2017.00445 PMC5581840

[bhy346C65] Ma Y, Tang C, Spetsieris PG, Dhawan V, Eidelberg D 2007 Abnormal metabolic network activity in Parkinson’s disease: test-retest reproducibility. J Cereb Blood Flow Metab. 27:597–605. 16804550 10.1038/sj.jcbfm.9600358 PMC4455600

[bhy346C66] Mallet N, Ballion B, Le Moine C, Gonon F 2006 Cortical inputs and GABA interneurons imbalance projection neurons in the striatum of parkinsonian rats. J Neurosci. 26:3875–3884. 16597742 10.1523/JNEUROSCI.4439-05.2006 PMC6674115

[bhy346C67] Mallet N, Pogosyan A, Sharott A, Csicsvari J, Bolam JP, Brown P, Magill PJ 2008 Disrupted dopamine transmission and the emergence of exaggerated beta oscillations in subthalamic nucleus and cerebral cortex. J Neurosci. 28:4795–4806. 18448656 10.1523/JNEUROSCI.0123-08.2008 PMC6670450

[bhy346C68] Martinu K, Monchi O 2013 Cortico-basal ganglia and cortico-cerebellar circuits in Parkinson’s disease: pathophysiology or compensation? Behav Neurosci. 127:222–236. 23244290 10.1037/a0031226

[bhy346C69] McCormick DA, Connors BW, Lighthall JW, Prince DA 1985 Comparative electrophysiology of pyramidal and sparsely spiny stellate neurons of the neocortex. J Neurophysiol. 54:782–806. 2999347 10.1152/jn.1985.54.4.782

[bhy346C70] Meyer-Lohmann J, Hore J, Brooks VB 1977 Cerebellar participation in generation of prompt arm movements. J Neurophysiol. 40:1038–1050. 409807 10.1152/jn.1977.40.5.1038

[bhy346C71] Mihailoff GA 1993 Cerebellar nuclear projections from the basilar pontine nuclei and nucleus reticularis tegmenti pontis as demonstrated with PHA-L tracing in the rat. J Comp Neurol. 330:130–146. 8468400 10.1002/cne.903300111

[bhy346C72] Moers-Hornikx VM, Vles JS, Tan SK, Cox K, Hoogland G, Steinbusch WM, Temel Y 2011 Cerebellar nuclei are activated by high-frequency stimulation of the subthalamic nucleus. Neurosci Lett. 496:111–115. 21511005 10.1016/j.neulet.2011.03.094

[bhy346C73] Molnar GF, Sailer A, Gunraj CA, Lang AE, Lozano AM, Chen R 2004 Thalamic deep brain stimulation activates the cerebellothalamocortical pathway. Neurology. 63:907–909. 15365147 10.1212/01.wnl.0000137419.85535.c7

[bhy346C74] Mottolese C, Richard N, Harquel S, Szathmari A, Sirigu A, Desmurget M 2013 Mapping motor representations in the human cerebellum. Brain. 136:330–342. 22945964 10.1093/brain/aws186

[bhy346C75] Mure H, Hirano S, Tang CC, Isaias IU, Antonini A, Ma Y, Dhawan V, Eidelberg D 2011 Parkinson’s disease tremor-related metabolic network: characterization, progression, and treatment effects. Neuroimage. 54:1244–1253. 20851193 10.1016/j.neuroimage.2010.09.028 PMC2997135

[bhy346C76] Nashef A, Cohen O, Israel Z, Harel R, Prut Y 2018 Cerebellar shaping of motor cortical firing is correlated with timing of motor actions. Cell Rep. 23:1275–1285. 29719244 10.1016/j.celrep.2018.04.035

[bhy346C77] Necchi D, Soldani C, Ronchetti F, Bernocchi G, Scherini E 2004 MPTP-induced increase in c-Fos- and c-Jun-like immunoreactivity in the monkey cerebellum. Eur J Histochem. 48:385–392. 15718205

[bhy346C78] Ni Z, Pinto AD, Lang AE, Chen R 2010 Involvement of the cerebellothalamocortical pathway in Parkinson disease. Ann Neurol. 68:816–824. 21194152 10.1002/ana.22221

[bhy346C79] Palmer SJ, Li J, Wang ZJ, McKeown MJ 2010 Joint amplitude and connectivity compensatory mechanisms in Parkinson’s disease. Neuroscience. 166:1110–1118. 20074617 10.1016/j.neuroscience.2010.01.012

[bhy346C80] Parker PR, Lalive AL, Kreitzer AC 2016 Pathway-specific remodeling of thalamostriatal synapses in Parkinsonian mice. Neuron. 89:734–740. 26833136 10.1016/j.neuron.2015.12.038 PMC4760843

[bhy346C81] Pasquereau B, DeLong MR, Turner RS 2016 Primary motor cortex of the parkinsonian monkey: altered encoding of active movement. Brain. 139:127–143. 26490335 10.1093/brain/awv312 PMC4794619

[bhy346C82] Pasquereau B, Turner RS 2011 Primary motor cortex of the parkinsonian monkey: differential effects on the spontaneous activity of pyramidal tract-type neurons. Cereb Cortex. 21:1362–1378. 21045003 10.1093/cercor/bhq217 PMC3097989

[bhy346C83] Paz R, Pelletier JG, Bauer EP, Pare D 2006 Emotional enhancement of memory via amygdala-driven facilitation of rhinal interactions. Nat Neurosci. 9:1321–1329. 16964249 10.1038/nn1771

[bhy346C84] Pelisson D, Goffart L, Guillaume A 1998 Contribution of the rostral fastigial nucleus to the control of orienting gaze shifts in the head-unrestrained cat. J Neurophysiol. 80:1180–1196. 9744931 10.1152/jn.1998.80.3.1180

[bhy346C85] Peng S, Eidelberg D, Ma Y 2014 Brain network markers of abnormal cerebral glucose metabolism and blood flow in Parkinson’s disease. Neurosci Bull. 30:823–837. 25260798 10.1007/s12264-014-1472-x PMC4556355

[bhy346C86] Popa D, Spolidoro M, Proville RD, Guyon N, Belliveau L, Lena C 2013 Functional role of the cerebellum in gamma-band synchronization of the sensory and motor cortices. J Neurosci. 33:6552–6556. 23575852 10.1523/JNEUROSCI.5521-12.2013 PMC6619065

[bhy346C87] Proville RD, Spolidoro M, Guyon N, Dugue GP, Selimi F, Isope P, Popa D, Lena C 2014 Cerebellum involvement in cortical sensorimotor circuits for the control of voluntary movements. Nat Neurosci. 17:1233–1239. 25064850 10.1038/nn.3773

[bhy346C88] Raichle ME 2015 The brain’s default mode network. Annu Rev Neurosci. 38:433–447. 25938726 10.1146/annurev-neuro-071013-014030

[bhy346C89] Sarnaik R, Raman IM 2018 Control of voluntary and optogenetically perturbed locomotion by spike rate and timing of neurons of the mouse cerebellar nuclei. ELife. 7. pii: e29546. 10.7554/eLife.29546 PMC5902160 29659351

[bhy346C90] Sausbier M, Hu H, Arntz C, Feil S, Kamm S, Adelsberger H, Sausbier U, Sailer CA, Feil R, Hofmann F, et al 2004 Cerebellar ataxia and Purkinje cell dysfunction caused by Ca2+-activated K+ channel deficiency. Proc Natl Acad Sci USA. 101:9474–9478. 15194823 10.1073/pnas.0401702101 PMC439001

[bhy346C91] Schonewille M, Khosrovani S, Winkelman BH, Hoebeek FE, De Jeu MT, Larsen IM, Van der Burg J, Schmolesky MT, Frens MA, De Zeeuw CI 2006 Purkinje cells in awake behaving animals operate at the upstate membrane potential. Nat Neurosci. 9:459–461. author reply 461. 16568098 10.1038/nn0406-459

[bhy346C92] Sen S, Kawaguchi A, Truong Y, Lewis MM, Huang X 2010 Dynamic changes in cerebello-thalamo-cortical motor circuitry during progression of Parkinson’s disease. Neuroscience. 166:712–719. 20034546 10.1016/j.neuroscience.2009.12.036 PMC2852615

[bhy346C93] Shirota Y, Hamada M, Hanajima R, Terao Y, Matsumoto H, Ohminami S, Tsuji S, Ugawa Y 2010 Cerebellar dysfunction in progressive supranuclear palsy: a transcranial magnetic stimulation study. Mov Disord. 25:2413–2419. 20818672 10.1002/mds.23298

[bhy346C94] Spetsieris PG, Ko JH, Tang CC, Nazem A, Sako W, Peng S, Ma Y, Dhawan V, Eidelberg D 2015 Metabolic resting-state brain networks in health and disease. Proc Natl Acad Sci USA. 112:2563–2568. 25675473 10.1073/pnas.1411011112 PMC4345616

[bhy346C95] Surmeier DJ, Ding J, Day M, Wang Z, Shen W 2007 D1 and D2 dopamine-receptor modulation of striatal glutamatergic signaling in striatal medium spiny neurons. Trends Neurosci. 30:228–235. 17408758 10.1016/j.tins.2007.03.008

[bhy346C96] Sutton AC, O’Connor KA, Pilitsis JG, Shin DS 2015 Stimulation of the subthalamic nucleus engages the cerebellum for motor function in parkinsonian rats. Brain Struct Funct. 220:3595–3609. 25124274 10.1007/s00429-014-0876-8

[bhy346C97] Teune TM, van der Burg J, van der Moer J, Voogd J, Ruigrok TJ 2000 Topography of cerebellar nuclear projections to the brain stem in the rat. Prog Brain Res. 124:141–172. 10943123 10.1016/S0079-6123(00)24014-4

[bhy346C98] Timmermann L, Gross J, Dirks M, Volkmann J, Freund HJ, Schnitzler A 2003 The cerebral oscillatory network of parkinsonian resting tremor. Brain. 126:199–212. 12477707 10.1093/brain/awg022

[bhy346C99] Tsai PT, Hull C, Chu Y, Greene-Colozzi E, Sadowski AR, Leech JM, Steinberg J, Crawley JN, Regehr WG, Sahin M 2012 Autistic-like behaviour and cerebellar dysfunction in Purkinje cell Tsc1 mutant mice. Nature. 488:647–651. 22763451 10.1038/nature11310 PMC3615424

[bhy346C100] Ugawa Y, Day BL, Rothwell JC, Thompson PD, Merton PA, Marsden CD 1991 Modulation of motor cortical excitability by electrical stimulation over the cerebellum in man. J Physiol. 441:57–72. 1816387 10.1113/jphysiol.1991.sp018738 PMC1180185

[bhy346C101] Valls-Sole J, Valldeoriola F 2002 Neurophysiological correlate of clinical signs in Parkinson’s disease. Clin Neurophysiol. 113:792–805. 12048039 10.1016/s1388-2457(02)00080-9

[bhy346C102] Viaro R, Morari M, Franchi G 2011 Progressive motor cortex functional reorganization following 6-hydroxydopamine lesioning in rats. J Neurosci. 31:4544–4554. 21430155 10.1523/JNEUROSCI.5394-10.2011 PMC6622898

[bhy346C103] Vila M, Perier C, Feger J, Yelnik J, Faucheux B, Ruberg M, Raisman-Vozari R, Agid Y, Hirsch EC 2000 Evolution of changes in neuronal activity in the subthalamic nucleus of rats with unilateral lesion of the substantia nigra assessed by metabolic and electrophysiological measurements. Eur J Neurosci. 12:337–344. 10651888 10.1046/j.1460-9568.2000.00901.x

[bhy346C104] Vitale F, Mattei C, Capozzo A, Pietrantoni I, Mazzone P, Scarnati E 2016 Cholinergic excitation from the pedunculopontine tegmental nucleus to the dentate nucleus in the rat. Neuroscience. 317:12–22. 26762800 10.1016/j.neuroscience.2015.12.055

[bhy346C105] White JJ, Sillitoe RV 2017 Genetic silencing of olivocerebellar synapses causes dystonia-like behaviour in mice. Nat Commun. 8:14912. 28374839 10.1038/ncomms14912 PMC5382291

[bhy346C106] Wu T, Hallett M 2005 A functional MRI study of automatic movements in patients with Parkinson’s disease. Brain. 128:2250–2259. 15958505 10.1093/brain/awh569

[bhy346C107] Wu T, Hallett M 2013 The cerebellum in Parkinson’s disease. Brain. 136:696–709. 23404337 10.1093/brain/aws360 PMC7273201

[bhy346C108] Wu T, Wang L, Chen Y, Zhao C, Li K, Chan P 2009 Changes of functional connectivity of the motor network in the resting state in Parkinson’s disease. Neurosci Lett. 460:6–10. 19463891 10.1016/j.neulet.2009.05.046

[bhy346C109] Wu T, Wang L, Hallett M, Chen Y, Li K, Chan P 2011 Effective connectivity of brain networks during self-initiated movement in Parkinson’s disease. Neuroimage. 55:204–215. 21126588 10.1016/j.neuroimage.2010.11.074

[bhy346C110] Yartsev MM, Givon-Mayo R, Maller M, Donchin O 2009 Pausing purkinje cells in the cerebellum of the awake cat. Front Syst Neurosci. 3:2. 19390639 10.3389/neuro.06.002.2009 PMC2671936

[bhy346C111] Yu H, Sternad D, Corcos DM, Vaillancourt DE 2007 Role of hyperactive cerebellum and motor cortex in Parkinson’s disease. Neuroimage. 35:222–233. 17223579 10.1016/j.neuroimage.2006.11.047 PMC1853309

