# Supplementary material for: Functional Alteration of Cerebello–Cerebral Coupling in an Experimental Mouse Model of Parkinson’s Disease
Source: Cereb Cortex. 2019 Jan 31;29(4):1752–66. doi: 10.1093/cercor/bhy346 (PMC6418382; doi:10.1093/cercor/bhy346)
Supplement: Supplementary Data [file bhy346_supplementary_materials.zip › bhy346_Menardyetal2018_CerCor_supp_legends.docx]

**Supplementary Figure 1**: Histology. **A**: quantification of the loss of striatal tyrosine-hydroxylase immunoreactivity. For each animal, the ratio between fluorescence signal in the lesioned and the non-lesioned side is computed. **B**: Example images of striatal TH immunostaining. **C**: histological verification of electrode placement.

**Supplementary Figure 2**: comparison of the dependency of firing rate as a function of week post-surgery (sham or lesion), and spike shape. **A**: density distribution of spike peak-to-trough delay (density obtained with a 20 µs gaussian kernel). The dotted line indicates the limit used to distinguish cells with narrow spikes (on the left of the line) and broad spikes (right of the line), respectively studied on panels B and C. Inset show example spike shapes of the two sort, with the mean trace in black, and the standard deviation in gray. **B**: average firing rates for the narrow spikes. The number of cells available per point is indicated close to each value. The small number of cells for some points precludes a reliable statistical assessment of difference between the Sham and 6-OHDA groups. **C**: same as B for broad spikes. This graph is very close to the one displayed in figure 3B; changing the threshold between narrow and broad spikes failed to reveal a class of neuron with clearly different behavior than the main population.

**Supplementary Figure 3**: accelerometric profile at rest in the third week post-lesion. **A**: power spectrum of the norm of the acceleration during resting period in sham-operated animal; each line corresponds to an animal ; the bold line and gray shading correspond to the average and average±sem limits of the power spectra . Note the presence of two peaks in the low frequency band (<10Hz) and mid-frequency (~ 25Hz); the former may be related to the small movements associated with breathing and heart beat; the later could correspond to thermogenic shivering. **B**: Same as A for 6-OHDA lesioned animals. One animal (labeled by a red star) exhibited a large power in the 10-20Hz band which was absent in sham-operated animal. This animal is further studied in panels C and D. **C**: overall profile of a full recording session with the norm of the rotational velocity (top) and of the total acceleration (bottom) along recording time for the mouse labeled with a star in panel B. The orange background shading corresponds to periods of activity while the green shading corresponds to period of rests. Note in the second part of the session brief episodes of activity (below the threshold of active behavior; the immobility of the animal was verified in video recording); a zoom of one of these episodes is presented in panel D. **D**: total acceleration (top) and time-frequency analysis (bottom) of one episode of activation. Such episode could correspond to a form of tremor, but was only observed in one animal.

**Supplementary figure 4**: Supplementary analysis of figure 5. **A,B** : same as figure 5E when only trials were the animal is in a resting state based on accelerometric data. **C**: quantification of peak size in time frequency analysis for ECoG response in resting period (as in Fig 5F). **D,E** : same as figure 5E when only trials were the animal is in a active state based on accelerometric data. **F**: quantification of peak size in time frequency analysis for ECoG response in resting period (as in Fig 5F). **G**: quantification of peak parameters in the low frequency range (5-10Hz) searched in the 200ms following the end of the stimulation.
